# Methyltrimethoxysilane Vapor Deposition Strategy for Preparing Superelastic and Hydrophobic Flexible Polyurethane Foams

**DOI:** 10.3390/polym17212814

**Published:** 2025-10-22

**Authors:** Hongyu Feng, Haijing Ma, Tian Jing, Bohan Zhai, Yanyan Dong, Shaohua Jiang, Xiaoshuai Han

**Affiliations:** 1 Jiangsu Co-Innovation Center of Efficient Processing and Utilization of Forest Resources, International Innovation Center for Forest Chemicals and Materials, College of Materials Science and Engineering, Nanjing Forestry University, Nanjing 210037, China; hongyu_feng_lab@126.com (H.F.); mhj3219457796@163.com (H.M.); 2210402104@njfu.edu.cn (B.Z.); 2 Zhejiang Kaifeng New Material Limited by Share Ltd., Quzhou 324404, China; 19858018980@163.com; 3 Institute of Environment and Sustainable Development in Agriculture, Chinese Academy of Agricultural Sciences, Beijing 100081, China; dongyanyan@caas.cn

**Keywords:** flexible polyurethane foam, trimethoxymethylsilane, CVD method, hydrophobic, resilience

## Abstract

Flexible polyurethane foam (FPUF) is widely used in buffer protection, biomedical, and wearable fields due to its light weight, high resilience, and adjustable mechanical properties. However, the traditional water foaming system is often accompanied by bottleneck problems such as cyclic fatigue attenuation, insufficient thermal stability, and surface hydrophilicity while achieving low density. In this study, a dense Si-O-Si cross-linked layer was in situ constructed on the surface of the foam by systematically regulating the water content of the foaming agent (1.5~2.5 wt%) and coupling with methyltrimethoxysilane (MTMS) chemical vapor deposition. Experiments show that the foam foamed with 2 wt% water content still maintains 0.0466 MPa compressive strength and 0.0532 MPa compressive modulus (modulus loss is only 16.6%) after 500 cycles of compression at 90% strain after MTMS deposition. MTMS modification drives the surface wettability to change from hydrophilic (70.4°) to hydrophobic (128.7°), and significantly improves thermal stability (the carbon residue rate at 800 °C increased to 25.5%, an increase of 59.4%). This study not only improves the resilience, but also endows the FPUF surface with hydrophobicity and thermal protection ability, which provides the feasibility for its wide application.

## 1. Introduction

Flexible polyurethane foam (FPUF), a porous polymeric material, has garnered significant attention in recent years due to its broad application potential across various industrial and consumer sectors. Its unique open-cell structure, excellent elasticity, and tunable physical properties make it a vital functional material for use in cushioning, packaging, automotive seat support layers, biomedical gaskets, and flexible wearable devices. Specifically, in the automotive industry, FPUF is widely employed in seating and interior components, greatly enhancing ride comfort and safety. In the healthcare field, its favorable breathability and softness render it an ideal material for mattresses, wheelchair cushions, and postoperative rehabilitation accessories [1,2,3,4,5,6].

However, although water-blown foaming technology can effectively reduce material density and create interconnected pores that confer high breathability, excessive water content leads to increased CO_2_ generation and intensified bubble coalescence. This, in turn, causes thinning of the pore walls and a reduction in the structural strength. Moreover, while the polyurea segments formed by the reaction of water with isocyanate can increase hard segment content, an excess of water aggravates microphase separation and interfacial stress concentration, resulting in the deterioration of macroscopic mechanical properties, such as compressive strength and resilience [7,8]. Another critical limitation lies in the inherent chemical structure of FPUF, which contains numerous polar groups (e.g., urethane and urea bonds), imparting pronounced hydrophilicity. Under humid or water-immersion conditions, water infiltration not only induces swelling and structural softening but also accelerates hydrolytic degradation, significantly shortening the service life of the foam. Therefore, relying solely on adjusting the water content cannot simultaneously achieve low density, high resilience, and long-term durability, nor can it confer hydrophobicity or improve thermal stability [9,10,11]. Conventional modification approaches, such as physical blending of hydrophobic additives (e.g., silane coupling agents) or surface coating with waterproofing layers, often suffer from drawbacks such as foam structural damage or poor interfacial adhesion, leading to inadequate durability of the modified properties. In contrast, chemical vapor deposition (CVD) using methyltrimethoxysilane (MTMS) enables the formation of a conformal siloxane coating under mild conditions through hydrolysis and condensation reactions on the FPUF surface [12,13,14,15]. This coating forms both chemical bonds and a physical barrier on the pore walls, thereby enhancing overall material performance without compromising the porous microstructure [16]. As a central variable in the water-blown foaming system, the water content directly influences the amount of CO_2_ released, the evolution of the pore structure, and the distribution of hard segments, which collectively determine the compressive fatigue behavior of the foam [17,18,19].

In this study, we systematically varied the water content (1.5–2.5 wt%) in a foam system based on polyethylene glycol-400 (PEG-400) and polymeric methylene diphenyl diisocyanate (PMDI) to investigate its effects on mechanical properties. Furthermore, the MTMS-CVD process was employed to construct a nanoscale siloxane–polyurethane composite interface, aiming to achieve synergistic improvements in surface hydrophobicity, mechanical strength, and thermal stability. To comprehensively evaluate the modification effects, we characterized the micromorphology and chemical composition of the foams before and after modification using scanning electron microscopy (SEM), Fourier transform infrared spectroscopy (FTIR), and X-ray photoelectron spectroscopy (XPS). Compression cycling tests were conducted to assess mechanical durability and energy absorption efficiency. Water contact angle measurements were used to evaluate surface hydrophobicity, and thermogravimetric analysis (TGA) was employed to examine thermal decomposition behavior and stability enhancements. The ultimate goal of this work is to elucidate the synergy between water-blown foaming and CVD-based surface modification, establishing an integrated manufacturing strategy from foaming to functionalization. This study provides a theoretical and technical foundation for developing high-performance FPUF materials with integrated functionalities such as durable hydrophobicity, high strength–toughness, and heat resistance, promoting their applications in advanced packaging, automotive interiors, biomedical devices, and specialized protective equipment.

## 2. Experimental Section

### 2.1. Materials

Polymethylene polyphenyl isocyanate (PAPI, NCO% = 30.5~32.0%) was supplied by Yantai Wanhua Chemical Group Co., Ltd. (Yantai, China). Silicone surfactant (AK8805) was purchased from Changzhou ZhuoLianZhiChuang Polymer Material Technology Co., Ltd. (Changzhou, China). Dibutyltin dilaurate (DBDL), Triethanolamine (TEA), Polyethylene glycol-400 (PEG-400), and Trimethoxymethylsilane (MTMS, ≥80%) were acquired from Aladdin Biochemical Technology Co., Ltd. (Shanghai, China). The experiment used laboratory-made deionized water as a foaming agent.

### 2.2. Preparation of Flexible Polyurethane Foam

PEG-400, deionized water, AK8805, DBDL, and TEA were added to the reaction vessel in turn, and uniformly mixed using a high-speed disperser (2000 r/min, 5 min). Then the appropriate amount of PAPI was added and mixed evenly. The mold was quickly poured into the preheated mold, and immediately transferred to the oven (60 °C) for free foaming and maintained for 24 h to complete the curing process [20,21]. Although the open-cell content was not explicitly measured, the high resilience and minimal residual strain observed are characteristic of flexible PU foams with a predominantly open-cell structure, which is typical for water-blown systems. The foams prepared by adding different amounts of water (1.5%, 1.75%, 2%, 2.25%, and 2.5%) were named PUF1.5%, PUF1.75%, PUF2%, PUF2.25%, and PUF2.5%; the specific formula is shown in Table 1.

### 2.3. Preparation of PUF @ MTMS Composite Foam

**Figure 1:**
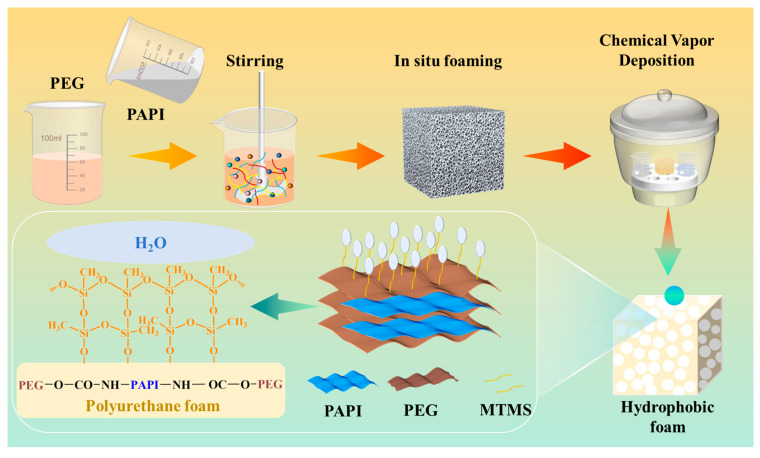
The schematic diagram of manufacturing strategy of PUF @ MTMS composite foam.

The composite foam was obtained byCVD. The PUF2% sample, which exhibited the optimal comprehensive mechanical performance, was selected for the MTMS deposition. Specifically, the foam, MTMS, and water in a mass ratio of 3:3:1 were put together into the dryer. Then, the dryer was sealed and vacuumized, and heated at 60 °C for 12 h. Finally, the composite foam was taken out and placed in a vacuum drying oven at 60 °C for further drying for 3 h to remove excess MTMS to obtain a composite foam PUF @ MTMS. The manufacturing strategy diagram and the chemical reactions during CVD are shown in Figure 1.

### 2.4. Characterization Methods

The microstructure of the foam samples was observed using a scanning electron microscope (SEM) (Phenom XL G2, Phenom-World BV, Eindhoven, The Netherlands), with elemental analysis performed by energy-dispersive spectroscopy (EDS) (X123FASTSDD, AMPTEK, Eindhoven, The Netherlands). Foam pore size was measured using the ImageJ software (ImageJ 1.54g). Fourier-transform infrared spectroscopy (FTIR) analysis of the solid foam was conducted using a VERTEX 80 V spectrometer (Bruker, Bremen, Germany) across the spectral range of 400–4000 cm^−1^. The cured samples underwent analysis using an X-ray photoelectron spectroscopy (XPS) spectrometer (Thermo Scientific K-Alpha, Waltham, MA, USA) with Al Kα radiation (hν = 1486.6 eV) at 150 eV for full-spectrum scanning and 50 eV for narrow-spectrum scanning, with resolutions of 1 and 0.1 eV, respectively. Mechanical properties were tested on a universal testing machine (UTM 500, Sansi Zongheng Technology Co., Ltd., Shenzhen, China). Apparent density measurement followed the GB/T 6343-1995 standard. Thermogravimetric analysis (TGA) was performed using a thermal analyzer (STA 449F3, NETZSCH, Selb, Germany), heating approximately 5 mg of cured powder sample from room temperature to 800 °C at 20 °C/min under a nitrogen atmosphere. Water contact angle (WCA) was measured by a contact goniometer (CA100D, Shanghai Precision Inno Precision Instrument Co., Ltd., Shanghai, China).

## 3. Characterization and Testing

### 3.1. Mechanical Properties of PUF

**Figure 2:**
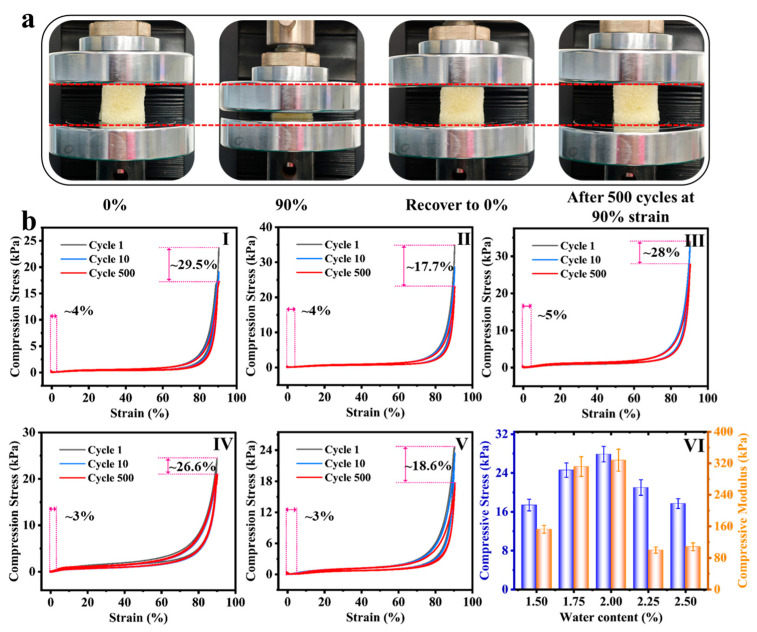
(**a**) Optical images of PUF under different compression conditions; (The red dotted line represents the height of the foam after compression under different conditions) (**b**) the cyclic compressive stress–strain curves after 1, 10, and 500 compression cycles of (**I**) PUF1.5%, (**II**) PUF1.75%, (**III**) PUF2%, (**IV**) PUF2.25%, (**V**) PUF2.5%, (**VI**) the compressive strength and compressive modulus of PUF after 500 compression cycles were measured.

In order to explore the influence mechanism of water content of foaming agent on the mechanical behavior of polyurethane foam (PUF), this study revealed the key rules by cyclic compression test combined with microstructure characterization. The comparison of the morphology of PUF before and after 500 compressions at 90% strain, shown in Figure 2a, confirms that the material has excellent elastic recovery characteristics, and the initial geometric configuration can be reproduced after any extrusion. The cyclic compression tests of foams prepared with different water contents (1.5~2.5%) at 90% strain (Figure 2b(I–V)) further show that all the samples still maintain excellent elasticity even after 500 times of high-strain compression, and the residual strain rate is only 3~5%. The regulation mechanism of water content on fatigue durability is systematically explained by Figure 2b(VI). When the water content increases from 1.5% to 2.0%, the increase in the amount of CO_2_ foaming leads to a decrease in density, and the simultaneous formation of urea bonds significantly thickens the pore wall and optimizes the distribution of hard segments, so that the cell skeleton maintains structural integrity after 500 cycles. Therefore, the component formula showed the highest residual compressive strength (27.9 kPa) and compressive modulus (329.7 kPa). However, when the water content increased to 2.25~2.5%, the excessive CO_2_ caused the excessive expansion and thinning of the pore wall, accompanied by local cell collapse and defect concentration, resulting in a significant attenuation of mechanical properties. The apparent density of the foams systematically decreased from 0.07 g/cm^3^ (PUF1.5%) to 0.051 g/cm^3^ (PUF2.5%) with increasing water content (Figure S1), as expected due to the increased volume of CO_2_ gas generated during foaming.

**Figure 3:**
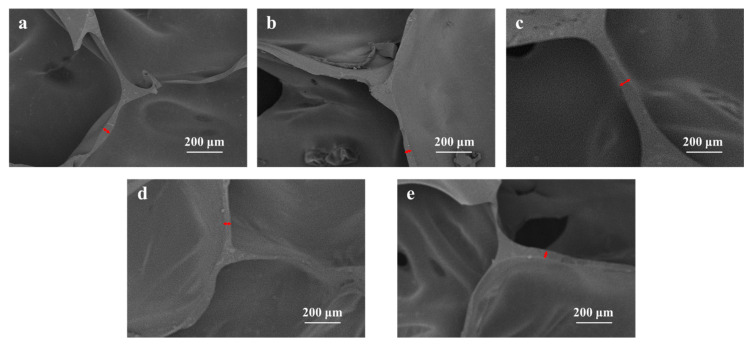
SEM micrographs of (**a**) PUF1.5%, (**b**) PUF1.75%, (**c**) PUF2%, (**d**) PUF2.25%, (**e**) PUF2.5%. (The red arrow indicates the thickness of the pore wall).

The SEM characterization (Figure 3) qualitatively shows a trend of decreasing pore wall thickness with increasing water content beyond 2.0 wt%, which correlates well with the observed attenuation of mechanical properties. This suggests that the structural integrity of the pore walls and struts, which is influenced by water content, is a critical factor for compression durability in these water-blown flexible foams. Quantitative analysis of the pore wall thickness further supports this trend (Figure S2). The cyclic compressive stress–strain curve and energy dissipation analysis (Figure S3) prove that the stress loss rate of the 2.0% water content sample is about 72% after 500 cycles, and the hysteresis loop area is the smallest, and the energy loss coefficient (0.28) is significantly lower than other components, indicating that the system has the best deformation reversibility and fatigue resistance [22,23].

### 3.2. Analysis of the Characterization Results of PUF @ MTMS

**Figure 4:**
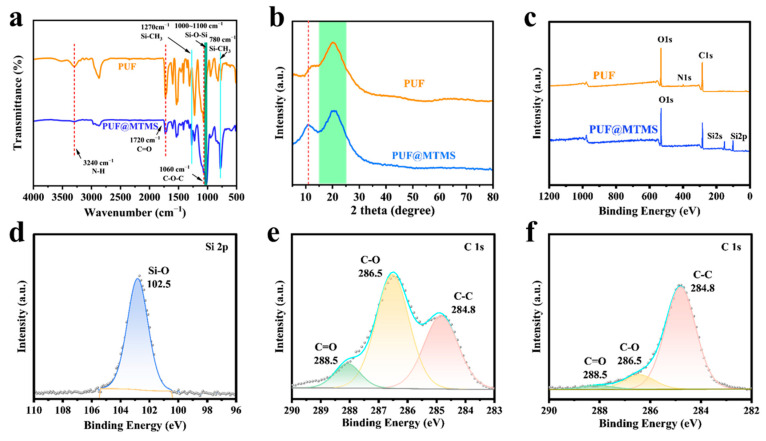
(**a**) FTIR curves and (**b**) XRD curves of PUF and PUF@MTMS. (**c**) Wide-scan XPS of PUF and PUF@MTMS. (**d**) Deconvoluted spectra of Si 2p peak of PUF@MTMS. (**e**) Deconvoluted spectra of C 1s peaks of PUF. (**f**) Deconvoluted spectra of C 1s peaks.

The successful deposition of the polysiloxane layer on the PUF skeleton was first quantitatively confirmed by measuring mass gain (Table S1). After gas phase treatment, the PUF samples showed measurable and consistent mass increase, with an average increase rate of 42.34%, which clearly indicated the adhesion of MTMS on the PUF scaffold. In order to clarify the reaction mechanism between MTMS and PUF, FTIR was used to analyze the evolution of functional groups on the surface of the foam before and after vapor deposition modification. As shown in Figure 4a, the original PUF and composite foam (PUF @ MTMS) showed polyurethane characteristic absorption peaks at 3240 cm^−1^ (N-H stretching vibration), 1720 cm^−1^ (C=O stretching vibration), and 1060 cm^−1^ (C-O-C stretching vibration) [24,25]. Different from the original foam, PUF @ MTMS shows an asymmetric stretching vibration peak of Si-O-Si in the region of 1000–1100 cm^−1^, and Si-CH_3_ symmetric deformation vibration is observed at 1270 cm^−1^ and 780 cm^−1^ [14,26]. The spectral evidence confirms that MTMS is chemically bonded to the polyurethane matrix, successfully grafting hydrophobic methyl groups and constructing a dense siloxane cross-linking layer, thereby endowing the PUF surface with a significant hydrophobic chemical microstructure. It can be seen from Figure 4b that the new diffraction peaks of polyurethane in the range of 10–15° after MTMS vapor deposition indicate that the polysiloxane network (-Si-O-Si-) formed during the treatment process establishes an ordered structure inside or at the interface of the material. This may be due to the layered stacking of MTMS hydrolysis condensation products or the formation of enhanced microphase separation of polyurethane hard segments induced by silicone components. The appearance of this ordered structure proves that MTMS successfully modified polyurethane without changing the amorphous nature of the matrix.

The surface chemical state of the sample was analyzed by XPS to further verify the reaction mechanism. It can be seen from Figure 4c that pure PUF can only see the characteristic peaks of C, N, and O elements belonging to the polyurethane structure. In contrast, PUF @ MTMS showed obvious Si 2s and Si 2p (Figure 4d) characteristic peaks at 153.9 eV and 102.9 eV, respectively, which should be attributed to the contribution of silicon in MTMS. In addition, the original obvious N 1s characteristic peak in PUF disappeared, indicating that the surface had been covered by the MTMS deposition layer, which confirmed the successful silanization of PUF. Figure 4e,f further show the high-resolution C 1s spectra of the sample surface before and after MTMS deposition and their peak fitting results. As shown in the figure, the C 1s spectrum of PUF can be deconvoluted into three components. The peak with a binding energy of 284.8 eV belongs to the C-C/C-H structure, the peak at 286.5 eV corresponds to the C-O bond, and the peak at 288.5 eV is derived from the C=O group. After MTMS deposition, the C 1s spectrum of PUF @ MTMS changed significantly: the relative area of C-C/C-H components increased significantly, while the relative intensity of C-O and C=O components decreased significantly, which was attributed to the introduction of methyl groups in MTMS molecules and their shielding effect on the signals of polar functional groups in PUF substrates. In summary, a polysiloxane-modified layer was successfully constructed on the surface of PUF by vapor deposition. Multi-dimensional evidence consistently confirmed the effective combination between MTMS and PUF matrix: mass gain test showed an average weight gain rate of 42.34%; the characteristic peaks of Si-O-Si (1000–1100 cm^−1^) and Si-CH_3_ (1270/780 cm^−1^) were detected by FTIR spectroscopy, which confirmed the chemical bonding. The XPS analysis showed that the characteristic peaks of silicon (Si 2s 153.9 eV, Si 2p 102.9 eV) and nitrogen signals disappeared, indicating that the PUF surface was completely covered by the MTMS deposition layer. In addition, the increase in the relative area of the C-C/C-H component and the decrease in the signal of oxygen-containing polar groups in the C 1s spectrum further verify the introduction of methyl and its shielding effect. These results together demonstrated that MTMS successfully achieved the hydrophobic modification of the matrix by forming a dense cross-linked polysiloxane layer on the surface of the PUF skeleton, and X-ray diffraction showed that the treatment process also induced the formation of an ordered structure.

**Figure 5:**
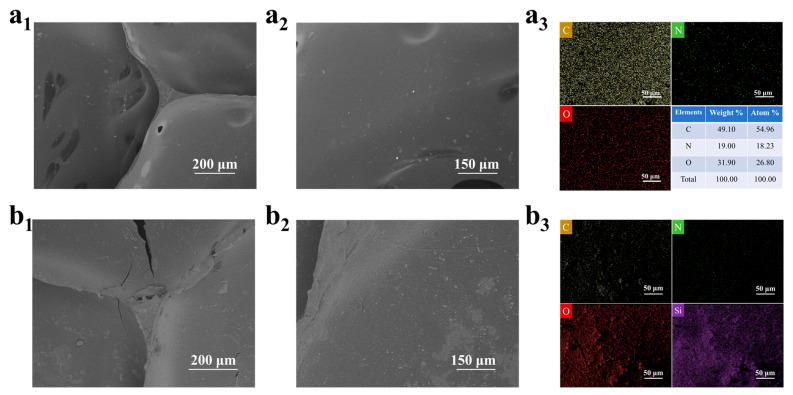
(**a_1_**,**a_2_**) SEM images of PUF; (**a_3_**) EDS spectra of C, O, and N elements in PUF; (**b_1_**,**b_2_**) SEM images of PUF @ MTMS; (**b_3_**) EDS spectra of C, O, N, and Si elements in PUF @ MTMS.

Based on the characterization analysis of SEM and EDS, the surface reconstruction mechanism of MTMS vapor deposition on PUF was revealed: the surface of the original PUF showed a smooth open pore structure (Figure 5a_1_,a_2_), the pore wall was uniform, and the EDS spectrum only detected C, O, and N elements (Figure 5a_3_); after MTMS vapor deposition, a dense granular siloxane deposition layer was formed on the surface of the foam skeleton (Figure 5b_1_,b_2_), and the uniform distribution of strong Si element was detected in the EDS spectrum (Figure 5b_3_), which confirmed that MTMS in situ constructed a continuous Si-O-Si cross-linking network on the surface of PUF through hydrolysis–condensation reaction [14]. In addition, the open pore structure of the foam can be seen in the optical microscope image (Figure S3), and the smooth surface of the PUF and the rough surface of the PUF @ MTMS.

This process involves a dual mechanism of chemical bonding (in which silanols can react with polyamino/hydroxyl groups to potentially form Si-O-C/Si-O-N covalent bonds) and physical coatings [20,21], synergistically endowing the foam surface with triple functions: (1) micro-nano roughening synergistically with low-surface-energy methyl significantly improves hydrophobicity (contact angle > 90°); (2) the siloxane network enhances the rigidity of the pore structure and improves the compressive deformation resistance; (3) the covalent bonding interface ensures the long-term stability of the coating. This process provides an efficient strategy for the surface functionalization of flexible porous materials through the synergistic path of chemical bonding–physical coverage.

### 3.3. Hydrophobicity of PUF @ MTMS

**Figure 6:**
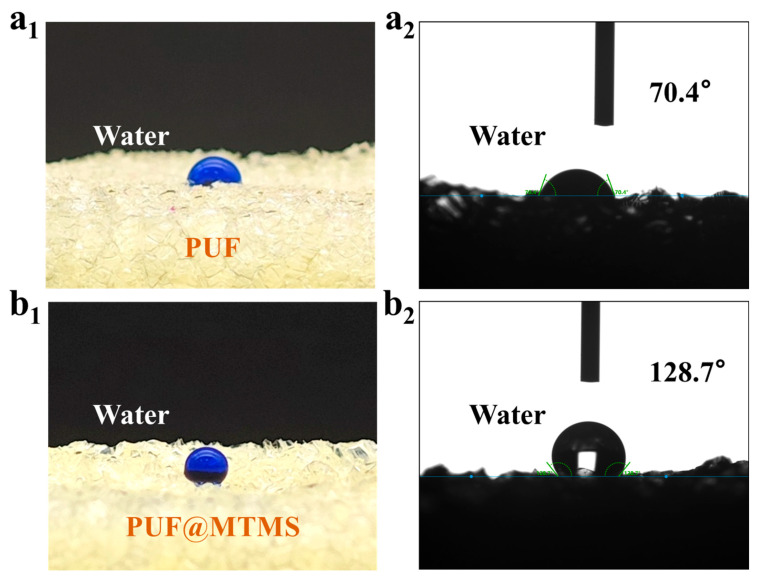
(**a_1_**) Optical images of the dyed water on the PUF; (**a_2_**) hydrophobic angles of PUF; (**b_1_**) optical images of the dyed water on the PUF @ MTMS; (**b_2_**) hydrophobic angles of PUF @ MTMS.

As a promising material, the hydrophilicity of polyurethane foam can restrict its practical application. Therefore, the hydrophobicity of PUF was enhanced through the use of MTMS via the CVD process. To evaluate the hydrophobicity of PUF, contact angle tests were conducted. Figure 6 shows images of the WCA tests on the surfaces of PUF and PUF @ MTMS. The results indicate that PUF exhibits high hydrophilicity, with a contact angle of 70.4°. In contrast, the WCA of PUF @ MTMS rises to 128.7°, marking a qualitative change towards a hydrophobic state. This transformation is attributed to the dual cooperative mechanism constructed by MTMS on the material surface—the low-surface-energy methyl groups (-CH_3_) introduced by the siloxane network effectively reduce interfacial tension [27,28,29], while the micro- and nano-scale rough structures generated by vapor deposition (as confirmed by SEM) trigger the Wenzel–Cassie wetting state transition by increasing the solid–gas interface proportion [30,31,32]. These two effects work synergistically to drive the contact angle across the hydrophilic–hydrophobic threshold (90°). The stable hydrophobic property of 128.7° not only confirms the successful reaction between polyurethane and MTMS but also provides a crucial surface performance foundation for the application of polyurethane foam in fields such as waterproof coatings, anti-biofouling, and liquid separation membranes.

### 3.4. Mechanical Properties and Thermal Stability of PUF @ MTMS

**Figure 7:**
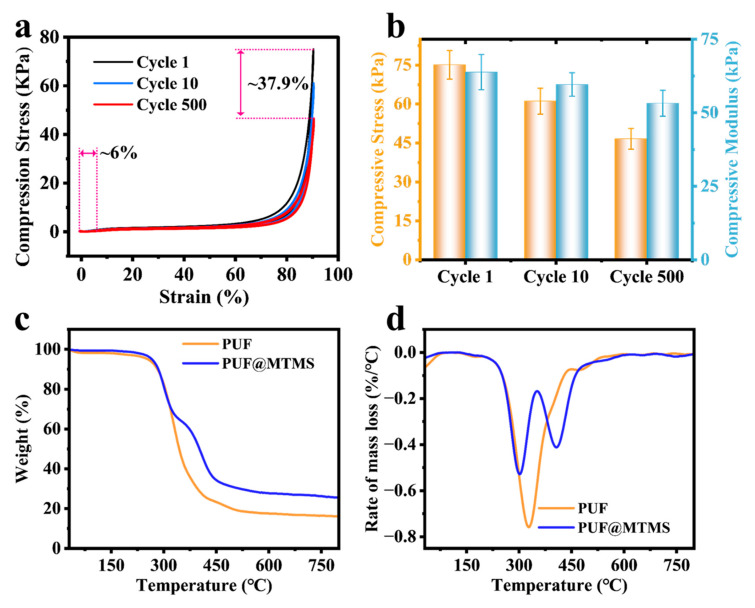
(**a**) The cyclic compressive stress–strain curves after 1, 10, and 500 compression cycles of PUF @ MTMS; (**b**) the compressive strength and compressive modulus of PUF @ MTMS. (**c**) TGA thermal decomposition curve and (**d**) DTG thermal decomposition curve of PUF and PUF @ MTMS.

The cyclic compression behavior of MTMS vapor-deposited polyurethane foam (Figure 7a) exhibits excellent mechanical stability. After 500 cycles of compression under 90% high-strain conditions, only about 6% of the strain loss and 62.1% of the stress retention rate are generated, indicating that the material has excellent deformation recovery and fatigue resistance. The evolution of the specific mechanical parameters shown in Figure 7b reveals the key mechanism: the initial compressive strength is 75.1 kPa, the modulus is 63.8 kPa, and the initial rigidity is characterized. After 10 cycles, the strength decreased to 61.1 kPa (attenuation of 18.6%), and the modulus decreased slightly to 59.6 kPa (attenuation of 6.6%), reflecting that the siloxane coating buffered stress concentration through microcrack energy dissipation. After 500 cycles, the strength was retained at 46.6 kPa (cumulative attenuation of 37.8%), and the modulus was maintained at 53.2 kPa (cumulative attenuation of 16.6%), showing a lower fatigue decay rate. This phenomenon is attributed to the dual synergistic mechanism of MTMS construction: the surface siloxane network acts as a rigid armor to inhibit cell collapse, and its covalent bonding with the polyurethane matrix (Si-O-C/Si-O-N) delays crack propagation through interfacial stress transfer, thereby synergistically achieving modulus stability under high cycles. This performance index confirms that MTMS modification not only improves surface hydrophobicity, but also provides a key mechanical guarantee for polyurethane foams in engineering scenarios that require long-term dynamic loading (such as shock absorption pads or medical implant buffer layers).

Based on TGA and differential thermogravimetric (DTG) curves (Figure 7c,d), it was found that vapor deposition of MTMS significantly regulated the thermal degradation pathway of PUF. The original PUF showed a typical two-stage degradation process: the first stage (210–448 °C) was mainly attributed to the breakage of ether bonds and carbamate bonds in the soft segment; the second stage (448–530 °C) corresponded to the aromatization reaction of the hard segment and the decomposition of the isocyanurate structure [33,34,35,36]. It is worth noting that the main degradation stage of PUF @ MTMS after MTMS vapor deposition was changed to 210–353 °C and 353–482 °C. During the initial degradation stage (210–353 °C), the Si-C and Si-O bonds of the oligomeric siloxane component were broken, generating volatile siloxane fragments accompanied by an endothermic process. Subsequently, in the second stage (353–482 °C), the fractured siloxane underwent a cross-linking reaction to form a dense Si-O-Si network barrier [27,37,38]. The thermally stable siliceous layer effectively inhibited the escape of internal combustible gas and the further thermal cracking of the polymer matrix. In addition, MTMS deposition significantly increased the high-temperature carbon residue rate of PUF, and the carbon residue at 800 °C increased from 16% of the untreated sample to 25.5%, further confirming the significant enhancement of its thermal stability.

## 4. Conclusions

In this study, PEG-400 and PAPI were used as the matrix, and the effect of water content in the blowing agent (1.5~2.5 wt%) on the mechanical properties of polyurethane was revealed by systematically adjusting the water content of the foaming agent. The siloxane–polyurethane interface was further constructed by CVD of MTMS. The results show that the foam with 2 wt% water content still maintains a compressive strength of 0.0279 MPa and a compressive modulus of 0.3297 MPa after 500 cycles at 90% strain, and the stress loss rate is only 28%. The corresponding pore wall thickness is positively correlated with mechanical properties, which verifies the pore wall toughening mechanism. After MTMS vapor deposition, a dense Si-O-Si cross-linked layer was formed in situ on the surface of the foam, which changed the surface from hydrophilic (70.4°) to hydrophobic (128.7°), and significantly improved the cycle stability: after 500 compressions, the compressive strength was 0.0466 MPa, the compressive modulus was 0.0532 MPa, the strain loss was reduced to 6%, and the stress retention rate was 62.1%. Thermogravimetric analysis confirmed that the siloxane network formed a dense barrier at high temperature, which increased the carbon residue rate from 16% to 25.5% at 800 °C. The water content–microstructure–macroscopic performance coupling law and CVD silanization strategy provide more ideas for the application of flexible polyurethane foam in multi-functional engineering scenarios such as dynamic load-bearing, waterproof and thermal insulation, and flame retardant buffering.

## Figures and Tables

**Table 1:** The specific formula of PUF.

Sample	PEG-400 (g)	Water (g)	AK-8805 (g)	DBDL (g)	TEA (g)	PAPI (g)
W_1.5_	100	1.5	2	1.125	0.375	69.48
W_1.75_	100	1.75	2	1.125	0.375	72.20
W_2_	100	2	2	1.125	0.375	74.92
W_2.25_	100	2.25	2	1.125	0.375	77.67
W_2.5_	100	2.5	2	1.125	0.375	80.37

## Data Availability

The data presented in this study are available upon request from the corresponding author. The data are not publicly available due to privacy restrictions.

## Supplementary Materials

The following supporting information can be downloaded at: https://www.mdpi.com/article/10.3390/polym17212814/s1, Figure S1: Apparent density of PUF1.5%, PUF1.75%, PUF2%, PUF2.25%, PUF2.5%; Figure S2: Foam wall thickness of (a) PUF1.5%, (b) PUF1.75%, (c) PUF2%, (d) PUF2.25%, (e) PUF2.5%, and (f) Summary of foam wall thickness of all PUFs; Figure S3: The energy loss coefficient and stress retention rate after 500 cycles of tests at a constant strain of 90%; Figure S4: The energy loss coefficient and stress retention rate of PUF @ MTMS were tested after 1,10 and 500 cycles at 90% constant strain; Figure S5: Optical microscope images of PUF and PUF @ MTMS. Table S1: The mass and density of PUF and PUF @ MTMS.
