# Supplementary material for: Methyltrimethoxysilane Vapor Deposition Strategy for Preparing Superelastic and Hydrophobic Flexible Polyurethane Foams
Source: Polymers (Basel). 2025 Oct 22;17(21):2814. doi: 10.3390/polym17212814 (PMC12608257; doi:10.3390/polym17212814)
Supplement: Supplementary file 1 [file polymers-17-02814-s001.zip › polymers-3874022-supplementary.pdf]

Supplementary

Methyltrimethoxysilane Vapor Deposition Strategy for Preparing Superelastic and Hydrophobic Flexible Polyurethane Foams

Hongyu Feng [1], Haijing Ma [1], Tian Jing [2], Bohan Zhai [1], Yanyan Dong [3], Shaohua Jiang [1,*] and Xiaoshuai Han [1,*]

[1] Jiangsu Co-Innovation Center of Efficient Processing and Utilization of Forest Resources, International
Innovation Center for Forest Chemicals and Materials, College of Materials Science and Engineering,
Nanjing Forestry University, Nanjing 210037, China; hongyu_feng_lab@126.com (H.F.); mhj3219457796@163.com (H.M.); 2210402104@njfu.edu.cn (B.Z.)
[2] Zhejiang Kaifeng New Material Limited by Share Ltd., Quzhou 324404, China; 19858018980@163.com
[3] Institute of Environment and Sustainable Development in Agriculture, Chinese Academy of Agricultural Sciences, Beijing 100081, China; dongyanyan@caas.cn
* Correspondence: shaohua.jiang@njfu.edu.cn (S.J.); xiaoshuai.han@njfu.edu.cn (X.H.)

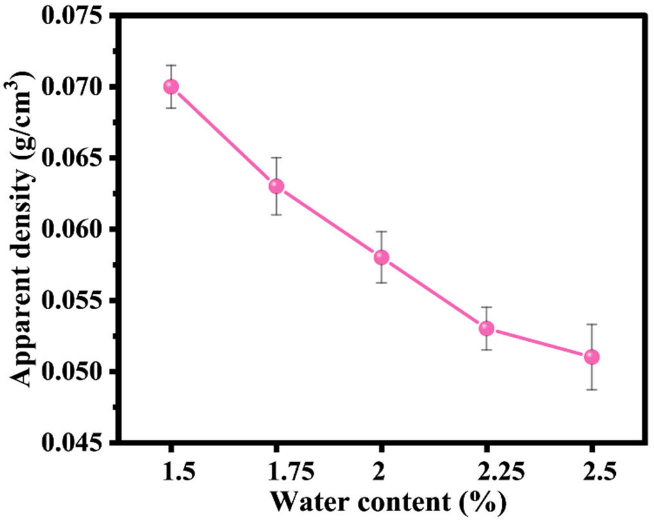

Figure S1. Apparent density of PUF1.5%, PUF1.75%, PUF2%, PUF2.25%, PUF2.5%.

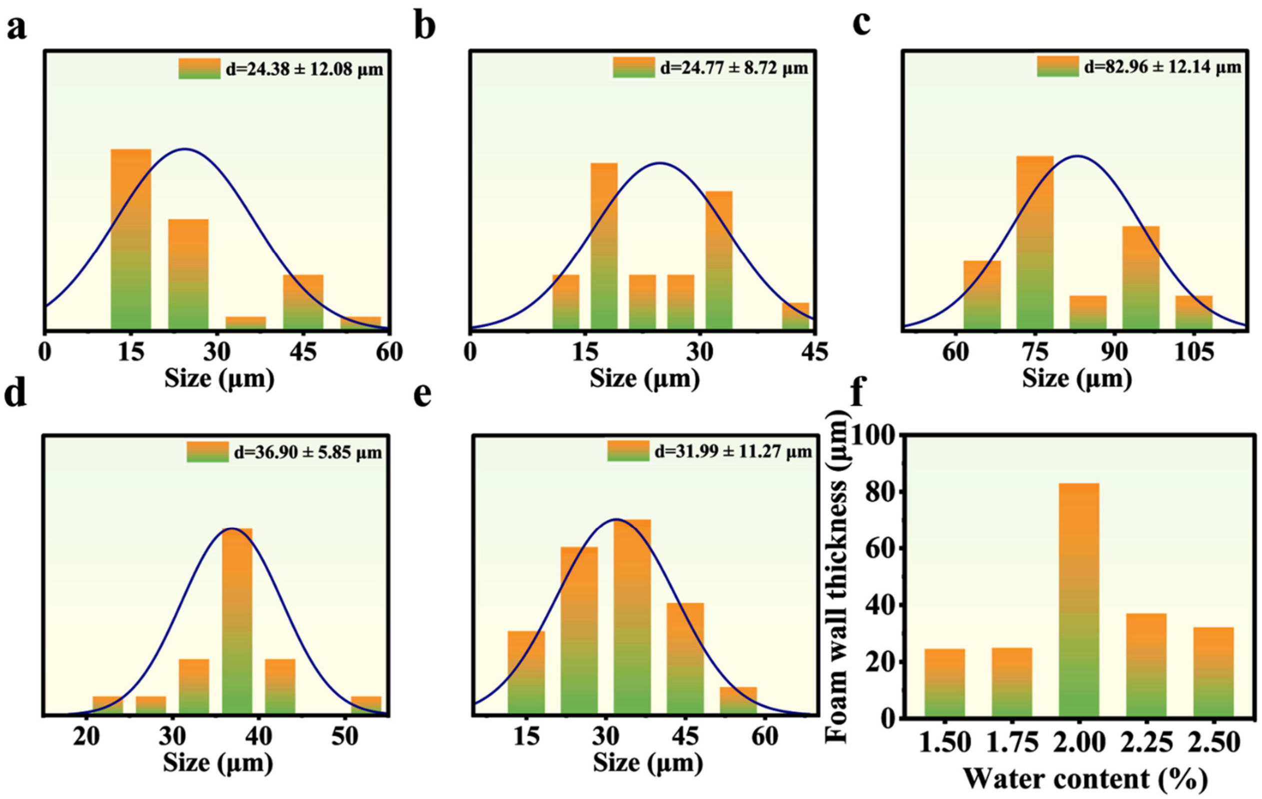

Figure S2. Foam wall thickness of (a) PUF1.5%, (b) PUF1.75%, (c) PUF2%, (d) PUF2.25%, (e) PUF2.5%, and (f) Summary of foam wall thickness of all PUFs.

Table S1. The mass and density of PUF and PUF @ MTMS.

Sample	W_0 (g)	Density-Before (g/cm³)	W_{CVD} (g)	Density-After (g/cm3)	Mass-gain (%)
I	2.1569	0.058	3.0749	0.0629	42.56
II	2.1819	0.056	3.1094	0.0615	42.60
III	2.5836	0.061	3.6651	0.0650	42.34

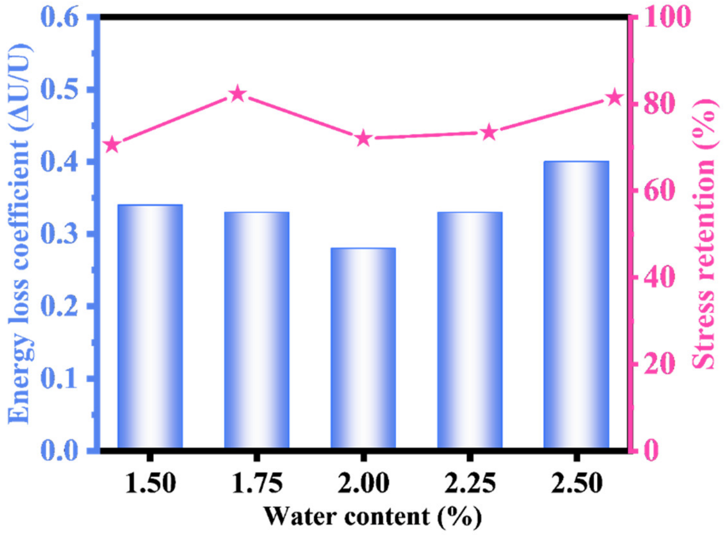

Figure S3. The energy loss coefficient and stress retention rate after 500 cycles of tests at a constant strain of 90%.

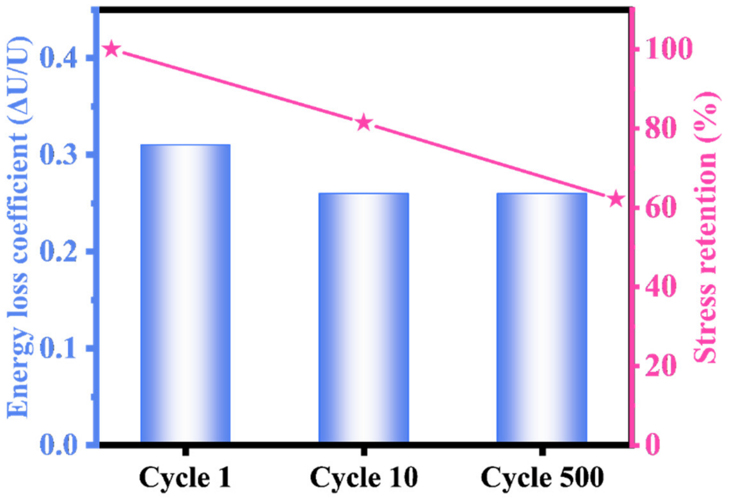

Figure S4. The energy loss coefficient and stress retention rate of PUF @ MTMS were tested after 1,10 and 500 cycles at 90 % constant strain.

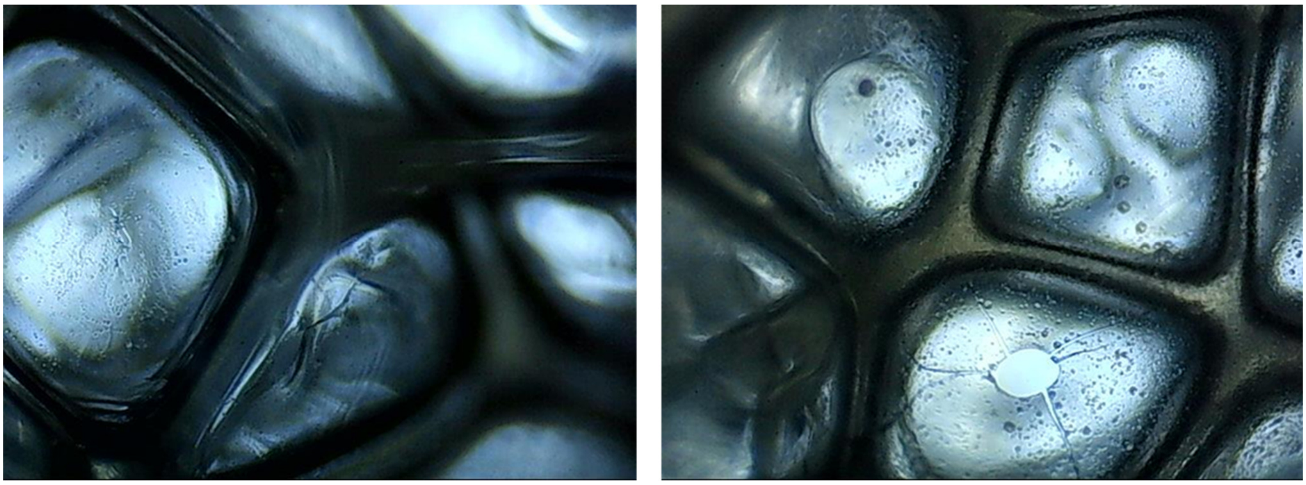

Figure S5. Optical microscope images of PUF and PUF @ MTMS.
